# The motivations and reputational consequences of spreading conspiracy theories

**DOI:** 10.1111/bjso.12784

**Published:** 2024-07-06

**Authors:** Shen Cao, Jan‐Willem van Prooijen, Mark van Vugt

**Affiliations:** ^1^ Department of Experimental and Applied Psychology Vrije Universiteit Amsterdam Amsterdam The Netherlands

**Keywords:** conspiracy theory, evolutionary psychology, reputation, the Adaptive‐Conspiracism Hypothesis, trait impression

## Abstract

Some people deliberately spread conspiracy theories. What are the reputational benefits and costs of doing so? The Adaptive‐Conspiracism hypothesis proposes that it pays to be vigilant against possible conspiracies, especially in case of intergroup threat. Those who spread conspiracy theories may therefore be seen as valuable group members. Few studies have focused on the reputational impact of spreading a conspiracy theory. We conducted five studies (*N*_Pilot_ = 303; *N*_Study1_ = 388; *N*_Study2_ = 560; *N*_Study3_ = 391; *N*_Study4_ = 373) where participants rated a conspiracy spreader (vs. a neutral person) on a range of personality traits in different intergroup contexts. The results indicated that conspiracy spreaders were consistently perceived as more dominant and less warm than people making non‐conspiratorial claims about certain events. Moreover, intergroup conflict attenuated the negative effects of spreading conspiracy theories on competence and warmth. These findings support the notion that besides drawbacks, spreading conspiracy theories can have benefits for the spreader's reputation, particularly during an intergroup conflict.

## INTRODUCTION

During the 2016 US presidential elections, Donald Trump propagated various conspiracy theories about his opponent Hillary Clinton. This trend persisted throughout his presidency, which has resonated well with his supporters. Following the 2020 elections, Trump's allegations that the presidential elections were stolen by Joe Biden inspired some of his followers to storm the U.S. Capitol on January 6th, 2021. Spreading conspiracy theories did not hinder Trump's successful political career. Trump is not alone in using conspiracy theories to gain support. Various politicians, online influencers and anti‐vaccination proponents use them to mobilize audiences (Chigwedere et al., 2008; Plenta, 2020). This observation raises questions about the reputational consequences of spreading conspiracy theories.

Conspiracy theories are defined as beliefs that a group of people meets in a secret agreement to achieve goals widely seen as malevolent (Bale, 2007). People may hold such beliefs privately, whereas others may share them publicly. Research primarily has highlighted negative perceptions of conspiracy believers, including gullibility and social rejection (Cassam, 2016; Lantian et al., 2018; Van Prooijen et al., 2023). Paradoxically, although being labeled a conspiracy theorist often incurs reputational damage and social exclusion, some people strategically spread conspiracy theories to boost popularity (Ren et al., 2022).

Grounded in evolutionary social psychology, our research examines the reputational implications of spreading conspiracy theories.^1^ We propose that spreading such theories enhances perceptions of dominance but decreases perceptions of warmth among perceivers. Additionally, intergroup conflict, a factor intensifying people's susceptibility to conspiracy theories, mitigates some of the negative reputational consequences associated with their spread.

### The adaptive conspiracism hypothesis

The Adaptive Conspiracism Hypothesis (ACH) posits conspiracy thinking as an adaptive response to ancestral coalitional violence (Van Prooijen & Van Vugt, 2018). Throughout history, conflicts between groups have led to coalitionary killings, shaping psychological mechanisms to guard against threats (Beckerman et al., 2009; Bowles, 2009; McDonald et al., 2012; Walker & Bailey, 2013), including out‐group prejudice and xenophobia (Neuberg & Schaller, 2016), strong in‐group identification (Van Vugt et al., 2007), support of strong leadership (Van Vugt & Spisak, 2008) and increased vigilance towards actual or presumed conspiracies. Conspiracies, hostile coalitions pursuing evil goals, posed significant threats in ancestral environments. A failure to detect dangerous conspiracies before they strike may have been a systematic cause of injury and reproductive loss (Van Prooijen & Van Vugt, 2018). A key implication of the ACH is that people become particularly susceptible to conspiracies during intergroup conflicts. Recognition of hostile conspiracies, even at a risk of false positives (cf. error‐management theory; Haselton & Nettle, 2006), may therefore have evolved as an adaptive response to the realistic dangers of intergroup conflict.

Ample evidence supports this assertion. Conspiracy beliefs are associated with individual predispositions connected to intergroup conflict, including authoritarianism (Abalakina‐Paap et al., 1999), social dominance orientation (Swami, 2012), prejudice (Imhoff & Bruder, 2014) and collective narcissism (Golec de Zavala & Federico, 2018; Sternisko et al., 2023). People high on these traits are more likely to perceive intergroup threat during conflict situations, increasing their susceptibility to conspiracy theories. Additionally, conspiracy theories are common among oppressed minority groups (Crocker et al., 1999; Van Prooijen et al., 2018), are used to demonize political opponents (e.g., Democrats vs. Republicans; Enders & Smallpage, 2016; Miller et al., 2016) and increase when outgroups are described as threatening (Mashuri & Zaduqisti, 2015). In football, supporters spread conspiracy theories on social media to explain their national team's loss (Bertin et al., 2022). At a macro‐level, conspiracy theories are more common in societies with higher conflict intensity (Hebel‐Sela et al., 2022) and may fuel a geo‐political conflict that is both non‐violent (e.g., the 2019 US–China trade war; Van Prooijen & Song, 2021) and violent (war; Pipes, 1997).

The dangers of encountering hostile coalitions and the corresponding link between conspiracy beliefs and intergroup conflict, suggest potential reputational benefits for people who spread conspiracy theories. Successfully exposing a conspiracy during dangerous conflicts may elevate one's status within the group, by contributing to group survival. As such, while spreading conspiracy theories may damage reputation in some circumstances (Lantian et al., 2018), it might benefit people's reputation during an acute intergroup conflict. Specifically, in competitive or openly hostile conditions, individuals may hold more positive personality impressions of people who spread conspiracy theories, viewing them as more effective leaders (Van Vugt, 2006).

### Personality impressions of conspiracy spreaders

Abundant literature in the social perception domain suggests a two‐dimensional representation that parsimoniously captures differences in person perception (Judd et al., 2005). This ranges from the dimensions of intellectual good/bad and social good/bad (Rosenberg et al., 1968), to the dimension of dominance (vs. submissive) and warmth or friendliness (vs. cold or hostility) in the Interpersonal Circumplex model (IC, Wiggins, 1979), to the dimension of competence and warmth in the Stereotype Content Model (SCM, Fiske et al., 2007). Despite varied terminology, the definitions of the two dimensions and the distinctions between dimensions are more similar than different (Judd et al., 2005). Interpersonal assessments revolve around discerning whether others' social intentions are beneficial (warmth) and evaluating their capability to execute intended actions (i.e., competence; Fiske et al., 2007).

People are also likely to evaluate conspiracy spreaders along these dimensions, influencing spreaders' social reputations. Thus, we measured perceived dominance, competence and warmth as the core variables in the present studies. Notably, although previous studies have suggested that the dominance and competence dimensions align closely across theories (Anderson & Kilduff, 2009; Judd et al., 2005; Oldmeadow & Fiske, 2007), we measured dominance and competence separately because dominance is especially associated with conflict and aggression, predicting winner‐loser effects in humans and nonhumans (Boehm, 2012; Maner, 2017; Preuschoft & Van Schaik, 2000). We therefore assume that perceived dominance is particularly relevant in the impressions people make about those who are spreading conspiracy theories.

A potential reputational effect is that conspiracy spreaders are perceived as dominant. The dominance (vs. submissive) dimension, indicative of status in IC (Wiggins, 1979), is defined as seeking mastery, power and individual differentiation (Wiggins, 1991). Dominant people are seen as forceful and assertive across multiple contexts (Buss & Craik, 1980) and successful in winning interpersonal conflicts (Preuschoft & Van Schaik, 2000). Dominance motivations are linked to conspiracy beliefs (Cichocka et al., 2022). For instance, Machiavellianism, a personality trait related to a manipulative and exploitative lifestyle, is positively correlated with a generic belief in conspiracy theories (Douglas & Sutton, 2011; March & Springer, 2019), which implies that conspiracy theories may help people attain dominance. Thus, we anticipated that people who spread conspiracy theories are perceived as dominant, especially during intergroup conflict. Paralleling the dominance effect, conspiracy spreaders may be seen as more suitable leaders but only under conflict conditions.

In addition, the perceived competence of conspiracy spreaders may increase because they demonstrate a talent for uncovering conspiracies. Competence is a dimension proposed in the SCM, which reflects traits related to perceived ability (e.g., intelligence, skill, creativity; Fiske et al., 2007). Perceived competence may correlate with perceived dominance because dominant people tend to succeed in competitions for status and power. For example, individuals higher in trait dominance were rated as more competent by observers, independent of their actual abilities (Anderson & Kilduff, 2009). We argue that the perceived competence of conspiracy spreaders increases, especially during intergroup conflict. Detecting conspiracies against one's group may position individuals as knowledgeable experts, providing benefits to ingroup members (Henrich & Gil‐White, 2001).

People who spread conspiracy theories may be perceived as less warm, because conspiracy theories communicate hostile attitudes towards members of other groups. The warmth dimension, which assesses people's social intentions such as friendliness and trustworthiness (Cuddy et al., 2008), is important for distinguishing friend from foe (Cuddy et al., 2011). Spreading conspiracy theories conveys qualities such as suspiciousness, paranoia and narcissism (Cichocka et al., 2022; van Prooijen et al., 2022), which may decrease perceived friendliness and trustworthiness. For example, studies found that people who actively defend conspiracy theories were expected to be socially excluded by observers (Lantian et al., 2018) and indeed, experienced more social rejection (Van Prooijen et al., 2023). However, such negative effects on perceived warmth may be attenuated during conflicts, as individuals may perceive conspiracy spreaders as warmer due to their perceived protective behavior toward the group, compared to in a no‐conflict situation.

Research on the reputational consequences of spreading conspiracy theories is limited. Green, Toribio‐Flórez, Douglas, Brunkow, and Sutton (2023) and Green, Toribio‐Flórez, and Douglas (2023) found that politicians, scientists and healthcare professionals supporting conspiracy theories were viewed as less trustworthy, competent and intelligent than those against conspiracy theories. These results do not contradict our theory which suggests that detecting conspiracies increases competence specifically during intergroup conflict. In the present research, we focused on how conspiracy detection was perceived by ingroup members. Namely, we tested whether being alert to potential conspiracies, especially during intergroup conflict, was perceived more positively (e.g., more competent) than not detecting conspiracies, extending previous research from an evolutionary perspective.

### The current research

The current research provides a different perspective by examining how people rate conspiracy spreaders on dominance, warmth and competence and by testing the moderating role of intergroup conflict in experimental designs. We propose that people perceive conspiracy spreaders as more dominant and more competent during intergroup conflicts. When conflicts are not salient, however, spreading conspiracy theories may harm people's reputation, especially in terms of warmth or competence.

The present research contains five studies. In a pilot study, we explored whether conspiracy spreaders (vs. neutral people) were perceived differently in terms of various personality traits while holding levels of intergroup conflict constant. Study 1 employed the same method but included established questionnaires to reliably measure dominance, competence and warmth. To test the contextual effect of conflicts, Studies 2–4 introduced different conditions (i.e., no‐conflict, resource conflict, physical conflict and cooperation) in both hunter‐gatherer and modern environments and tested participants' perceptions. Studies 1 to 4 were pre‐registered on the Open Science Framework (OSF).^2^ We also included exploratory analyses with no explicit hypothesis in the pre‐registration, involving variables such as individual differences (e.g. social class; social dominance orientation), which were not reported in this manuscript.

## PILOT STUDY

In the pilot study, we employed a trait‐impression method to measure personality traits. We hypothesized that in intergroup conflict condition, people perceived conspiracy spreaders as more dominant and competent, but less warm, than neutral individuals who convey no conspiracy message.

### Method

#### Participants

Three hundred and three participants were recruited from the US via the Prolific platform. Thirteen participants were excluded because they failed the manipulation check, resulting in 290 eligible participants (162 females, 122 males and 6 others). Their ages ranged from 18 to 75 years (*M* = 40.0, *SD* = 13.9). A sensitivity analysis via G*power (version 3.1; Faul et al., 2009) showed that the minimum effect size that can be reliably detected with this sample size is Cohen's *d* = 0.33, critical *t*(288) = 1.97, 80% power, *α* = .05. All power analyses were conduct via the same version of G*power.

#### Materials

**FIGURE 1:**
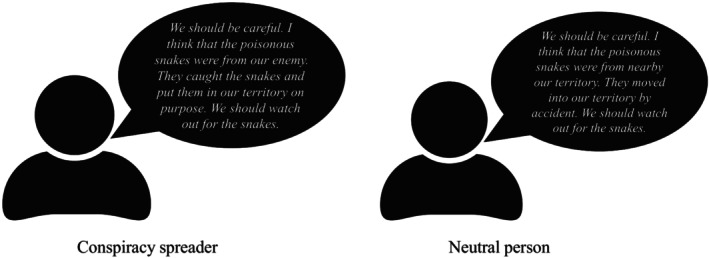
Conspiracy spreader vs. neutral person.

The detailed scenarios of all studies are available in the Online Supplementary Materials (OSM). Participants imagined that they were a member of a tribe living in the Amazon Rainforest, competing against another tribe. Recently, some of their tribe members died of bites by poisonous snakes. In the conspiracy condition, a member called Aru said that the snakes were from their opponent while in the neutral condition Aru said that the snakes moved in by accident (Figure 1). The conspiracy scenario suggests that a group of individuals (‘the enemy’) engaged in secret actions (i.e., introducing poisonous snakes covertly) with the intent of achieving a malevolent goal (i.e., causing harm to members of the opposing tribe).

#### Measurements

Questions were adapted from The Eight‐Factor Structure of Personality Traits (De Raad & Barelds, 2008). We chose 6 dimensions that were related to our predictions: Virtue, Competence, Agreeableness, Conscientiousness, Hedonism and Conventionality. Each dimension included three descriptions (e.g., ‘a friendly person’) rated on a 7‐point Likert scale (1‐ ‘strongly disagree’ to 7‐ ‘strongly agree’).

#### Procedure

Participants were randomly assigned to either the neutral condition or the conspiracy condition. After reading the scenario participants responded to the trait‐impression questions and a manipulation check. There are two attention‐check questions asking participants to select a specific answer, as well as a manipulation check asking participants to choose what Aru proclaimed. All the studies were approved by The Scientific and Ethical Review Board.

### Results

#### Descriptive results

The matrix of means, standard deviations and correlations of all measured items are in the OSM.

#### Exploratory factor analysis and an independent‐sample *t*‐test of the original items

To test whether the 18 items align with our theory, we conducted an exploratory factor analysis (EFA). The detailed results are shown in the OSM. In the conspiracy condition, Aru was perceived as more dominant, complicated, unique, impulsive and thought to rouse public sentiment. However, they thought of Aru as less friendly, trustworthy, sympathetic, goodhearted, helpful and careful. The EFA suggested a two‐factor‐model: Factor 1 was loaded by 12 items (e.g., friendly, trustworthy, sympathetic) related to warmth. Factor 2 was loaded by 6 items related to dominance (e.g., going along with everything, dominant and rousing public sentiment). Although we did not find a factor that represented competence, the results were partially in line with our predictions.

#### Independent‐sample *t*‐test of the two factors

We averaged the items that loaded on each factor in the EFA and conducted another independent‐sample *t*‐test. The results suggested that conspiracy spreaders scored lower on warmth (Factor 1, *M* = 4.6, *SD* = 0.9) compared to neutral people, *M* = 5.1, *SD* = 0.7, *t*(288) = 5.36, *p* < .001, *d* = −0.63, CI_95%_ [−0.87, −0.40]. Moreover, they were perceived as more dominant (Factor 2, *M* = 4.8, *SD* = 0.7) than neutral people, *M* = 4.1, *SD* = 0.7, *t*(288) = −8.11, *p* < .001, *d* = 0.96, CI_95%_ [0.71, 1.20]. These results were consistent with our predictions.

### Discussion

The pilot study found that conspiracy spreaders were considered more dominant and less warm than neutral people, while keeping intergroup conflict constant. To replicate and extend the results, Study 1 employed more reliable measurements of trait impressions of dominance, competence and warmth.

## STUDY 1

Study 1 employed the same methodology with reliable measurements. Additionally, we measured leadership qualities and commitment to the group. Conspiracy spreaders may also be viewed as leader‐like because dominant people convey the capability of managing conflicts and people prefer them for leaders in intergroup conflicts (Van Vugt & Grabo, 2015). Moreover, since exposing conspiracies could protect ingroup members from potential threats, conspiracy spreaders might be thought to dedicate themselves to their group. We did not have a specific prediction about commitment, however.

Altogether, in Study 1, we hypothesized that people perceive conspiracy spreaders as more dominant and leader‐like (H1.1), more competent (H1.2) and less warm (H1.3) than neutral people.

### Method

#### Participants

To achieve 80% power (*d* = .30, *α* = .05), a minimum of 352 participants are required. To be on the safe side, 401 participants were recruited via the Prolific platform. Participants were from the US whose first language was English. Because 13 participants failed the manipulation check, the final sample contained 388 eligible participants including 193 males, 190 females and 5 others (18–79 years old; *M* = 37.3, *SD* = 12.3).

#### Materials

The scenarios used in Study 1 were the same as those in the pilot study.

#### Measurement

##### Perceived dominance

The dominance subscale from the Mini‐markers of Evil questionnaire (Harms et al., 2007; Kim et al., 2020) was selected. It included 5 items (e.g., ‘I think Aru is dominant/forceful’) measured by a 7‐point Likert scale (1‐ ‘strongly disagree’ to 7‐ ‘strongly agree’). The scale had good internal reliability (*α* = .86).

##### Perceived competence

The adapted version of The Self‐Competence Scale (5 items rated on a 7‐point Likert scale, Tafarodi & Swann, 1995) was used to measure perceived competence (e.g., ‘Aru has much potential’). The scale had good internal reliability (*α* = .86).

##### Perceived leader‐like qualities

Five items were selected from the Akron Leadership Questionnaire (on a 7‐point Likert scale, Lord et al., 1984) to measure perceived leader‐like qualities (e.g., ‘Aru coordinates groups’). The scale had good internal reliability (*α* = .77).

##### Perceived warmth

Five items from the Agreeableness subscale of the Big Five Inventory (on a 7‐point Likert scale, John & Srivastava, 1999) were used to measure perceived warmth (e.g., ‘I think that Aru is helpful and unselfish with others’). The scale had good internal reliability (*α* = .89).

##### Perceived commitment to the group

The Commitment subscale of the Self‐Categorization, Commitment to The Group and Group Self‐Esteem Scale were measured (5‐items on a 7‐point Likert scale, Ellemers et al., 1999; e.g., ‘Aru would like to work for your tribe.’). The scale had good internal reliability (*α* = .82).

#### Procedure

As in the pilot study, participants read the background story and the words Aru said (either conspiratorial or neutral). They then finished the trait‐impression questions.

### Results

#### Descriptive results

The means, standard deviations and correlations are shown in Table 1.

**TABLE 1:** The means, standard deviations and correlations of measured variables (*N* = 388).

	*M*	*SD*	1	2	3	4	5	6	7
1. Gender	1.5	0.5	–						
2. Age	37.3	12.3	.05	–					
3. Condition	0.5	0.5	<.01	.03	–				
4. Dominance	4.7	1.1	.05	.08	.25***	–			
5. Leaderlike	4.9	1.0	.06	.02	.07	.50***	–		
6. Competence	4.9	1.0	.06	.11*	−.03	.36***	.55***	–	
7. Warmth	4.5	1.2	.05	.04	−.44***	−.13**	.23***	.43***	–
8. Commitment	6.0	1.0	.04	.05	.04	.18***	.35***	.42***	.20***

*Note*: For Gender, ‘male’, ‘female’ and ‘other’ were coded as ‘1’, ‘2’ and ‘3’; for Condition, ‘0’ referred to ‘neutral’ while ‘1’ referred to ‘conspiracy’.

* *p* < .05.

** *p* < .01.

*** *p* < .001.

#### Hypotheses tests

**FIGURE 2:**
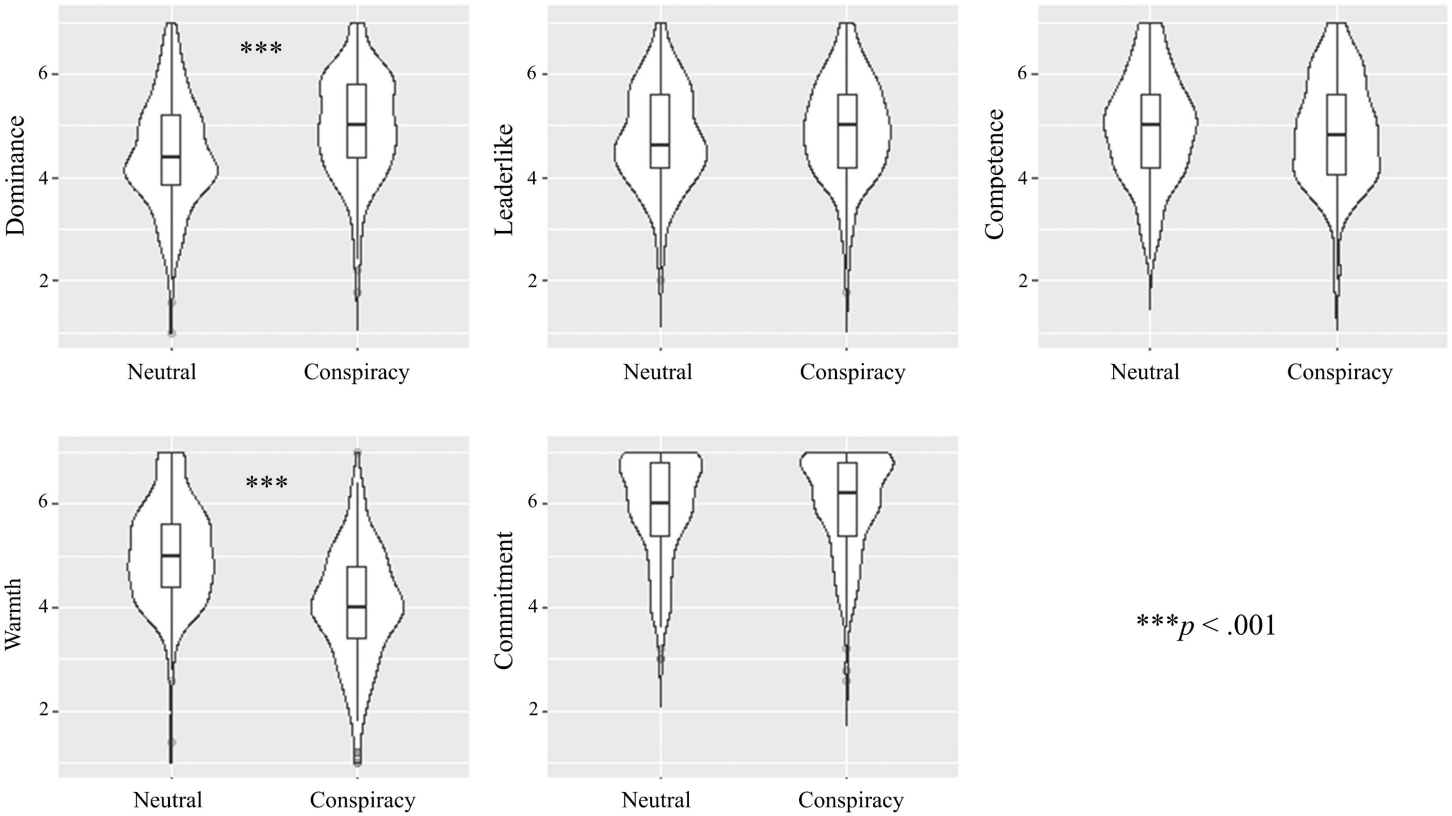
The results of the independent samples *t*‐tests.

Results from the independent‐sample *t*‐tests are presented in Figure 2. Consistent with our predictions, participants perceived the conspiracy spreader as more dominant (*M* = 5.0, *SD* = 1.0) and less warm (*M* = 4.0, *SD* = 1.1) than the neutral person, *M*_dominance_ = 4.5, *SD* = 1.1, *t*(386) = 5.10, *p* < .001, *d* = 0.52, CI_95%_ [0.32, 0.72]; *M*_warmth_ = 5.1, *SD* = 1.0, *t*(386) = −9.70, *p* < .001, *d* = −0.99, CI_95%_ [−1.20, −0.78]. However, no significant difference was found in perceived leader‐like qualities, *M*_conspiracy_ = 4.9, *SD* = 1.0; *M*_neutral_ = 4.8, *SD* = 1.0, *t*(386) = −1.46, *p* = .145, *d* = 0.15, CI_95%_ [−0.05, 0.35] and competence, *M*_conspiracy_ = 4.9, *SD* = 1.0; *M*_neutral_ = 5.0, *SD* = 1.0, *t*(386) = 0.60, *p* = .547, *d* = −0.06, CI_95%_ [−0.26, 0.14] between the neutral person and the conspiracy spreaders, which was not in line with our predictions. Besides, no significant difference in perceived commitment to the group, *M*_conspiracy_ = 6.0, *SD* = 1.0; *M*_neutral_ = 5.9, *SD* = 1.0, *t*(386) = −0.76, *p* = .450, *d* = 0.08, CI_95%_ [−0.12, 0.28] was found either.

### Discussion

Study 1 supported our predictions that people perceive conspiracy spreaders as more dominant (although not more leader‐like, H1.1) and less warm (H1.3) than neutral people when two groups were competing against each other. However, we did not find significant differences in competence between spreaders and neutral people, which was not in line with H1.2.

## STUDY 2

The pilot study and Study 1 held a relatively mild conflict constant (i.e., resource conflict which is an indirect threat to people's survival). In Study 2, we added a condition of a physical conflict (which is more directly life‐threatening) and a no‐conflict condition. We argue that a more life‐threatening situation may make conspiracy spreaders more valuable. We hypothesized that the conspiracy spreaders were perceived as (1) more dominant than the neutral person (H2.1), especially during a physical conflict (H2.2) and (2) less warm than the neutral person (H2.3). The results for perceived commitment were exploratory and are reported in the Supporting Information.

### Method

#### Participants

To achieve 80% power (a small‐to‐medium effect size for the interaction, *f* = .15, *α* = .05), a minimum of 432 participants were required.^3^ To be on the safe side, 600 participants from the U.S. were recruited (100 for each condition) via Prolific. Forty participants who failed the attention check and manipulation check were excluded. As a result, 560 participants were eligible (aged from 18 to 81, *M* = 42.3, *SD* = 13.5), including 277 males, 278 females and 5 others.

#### Materials

In addition to the resource conflict, a neutral scenario and a physical conflict scenario were developed. In the neutral context, the two tribes have no connection to each other while in the physical conflict context, the two tribes have a history of violence. The conspiracy theory and neutral claim were the same as in the pilot study and Study 1.

#### Measurements

All measurements were the same as in Study 1 and had good internal reliability: *α* (dominance) = .87; *α* (leadership qualities) = .81; *α* (competence) = .85; *α* (warmth) = .90.

#### Procedure

Participants read the texts and finished the trait‐impression questions.

### Results

#### Descriptive results

The means, standard deviations and correlations are shown in Table 2.

**TABLE 2:** The means, standard deviations and correlations of measured variables (*N* = 560)

	*M*	*SD*	1	2	3	4	5	6	7
1. Gender	1.5	0.5	–						
2. Age	42.3	13.5	.14***	–					
3. Narrative	0.5	0.5	.04	−.01	–				
4. Conflict	1.0	0.8	.04	−.03	.02	–			
5. Dominance	4.7	1.1	.08	.11*	.18***	.02	–		
6. Leaderlike	4.8	1.0	−.03	−.04	−.01	.05	.59***	–	
7. Competence	4.8	1.0	.04	< .01	−.18***	.06	.40***	.63***	–
8. Warmth	4.5	1.2	.03	.03	−.45***	.02	.06	.35***	.59***

*Note*: For Gender, ‘male’, ‘female’ and ‘other’ were coded as ‘1’, ‘2’ and ‘3’; For Narratives, ‘0’ referred to ‘neutral’ while ‘1’ referred to ‘conspiracy’; for Conflict, ‘0’ referred to ‘no‐conflict’ and ‘1’ referred to ‘resource conflict’ and ‘2’ referred to ‘physical conflict’.

* *p* < .05.

*** *p* < .001.

#### Hypothesis testing

**FIGURE 3:**
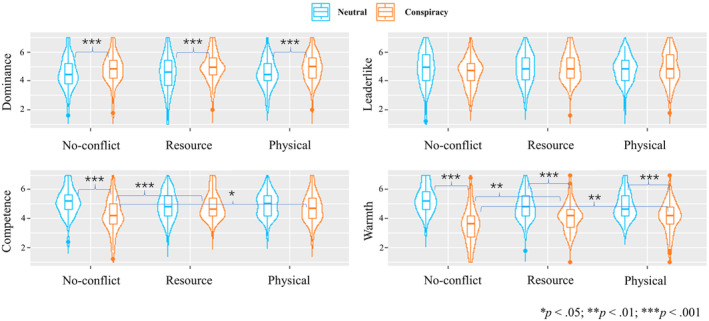
The results of the two‐way ANOVAs.

A 2 × 3 ANOVA was used to test the differences in dependent variables among six experimental conditions. The results of the 5 two‐way ANOVAs are displayed in Figure 3.

For perceived dominance, people considered the conspiracy spreaders as more dominant (*M* = 4.9, *SD* = 1.0) than neutral people (*M* = 4.5, *SD* = 1.1), *F*(1, 554) = 18.35, *p* < .001, $\eta_{p}^{2}$ = .03, CI_95%_ [0.01, 0.07], which was in line with H2.1. However, no interaction was found, *F*(2, 554) = 0.50, *p* = .606, $\eta_{p}^{2}$ < .01, CI_95%_ [0.00, 0.01] (H2.2 was not supported), suggesting that the effects of spreading conspiracy theories on dominance appear independent from intergroup conflict.

For perceived warmth, conspiracy spreaders (*M* = 3.9, *SD* = 1.1) were perceived as less warm than neutral people (*M* = 5.0, *SD* = 1.0) in all contexts, *F*(1, 554) = 148.51, *p* < .001, $\eta_{p}^{2}$ = .21, CI_95%_ [0.16, 0.27]. Besides, a significant interaction emerged, *F*(2, 554) = 14.71, *p* < .001, $\eta_{p}^{2}$ = .05, CI_95%_ [0.02, 0.09]. Multiple comparisons with Bonferroni adjustment suggested that the conspiracy spreaders were perceived as the least warm (*M* = 3.6, *SD* = 1.2) in the no‐conflict context. Their perceived warmth increased in resource (*M* = 4.1, *SD* = 1.0, *t* = −3.88, *p* = .002) and physical conflicts (*M* = 4.1, *SD* = 1.1, *t* = −3.93, *p* = .001). No difference between the two conflict conditions was found (*t* = −0.04, *p* = 1.000). We did not find significant differences in warmth among the neutral people in the no‐conflict condition (*M* = 5.3, *SD* = 0.9) versus the resource conflict condition (*M* = 4.9, *SD* = 1.0, *t* = 2.81, *p* = .076) and the physical conflict condition (*M* = 4.9, *SD* = 0.9, *t* = 2.71, *p* = .105).

For perceived competence, conspiracy spreaders (*M* = 4.6, *SD* = 1.0) were perceived as less competent than neutral people (*M* = 5.0, *SD* = 0.9), *F*(1, 554) = 18.93, *p* < .001, $\eta_{p}^{2}$ = .03, CI_95%_ [0.01, 0.07]. A significant interaction emerged, *F*(2, 554) = 10.09, *p* < .001, $\eta_{p}^{2}$ = .04, CI_95%_ [0.01, 0.07]. Multiple comparisons with Bonferroni adjustment suggested that in the no‐conflict context, conspiracy spreaders' competence (*M* = 4.3, *SD* = 1.2) was perceived as lower than the neutral person (*M* = 5.1, *SD* = 0.9, *t* = 6.08, *p* < .001). Spreaders' competence increased in both the resource (*M* = 4.9, *SD* = 0.9, *t* = −4.36, *p* < .001) and physical conflict conditions (*M* = 4.7, *SD* = 1.0, *t* = −3.36, *p* = .013), however. The neutral person's competence remained the same across the three contexts (*M*_resource_ = 4.9, *SD* = 0.9, *t* = 1.76, *p* = 1.000; *M*_physical_ = 4.9, *SD* = 0.9, *t* = 1.33, *p* = 1.000). The significant difference in perceived competence between the conspiracy spreader and neutral person was not found in the two conflict contexts, which was consistent with Study 1.

For leader‐like qualities, no main effect of conflict was found, *F*(2, 554) = 1.42, *p* = .243, $\eta_{p}^{2}$ = .01, CI_95%_ [0.00, 0.02], or narrative, *F*(1, 554) = 0.08, *p* = .772, $\eta_{p}^{2}$ < .01, CI_95%_ [0.00, 0.01]. No interaction was found, *F*(2, 554) = 1.38, *p* = .253, $\eta_{p}^{2}$ < .01, CI_95%_ [0.00, 0.02].

### Discussion

Study 2 replicated the findings of the pilot and Study 1, showing that conspiracy spreaders were perceived as more dominant (H2.1) and less warm (H2.3) than neutral people. While conflict intensity did not moderate the effects on dominance (H2.2 was not supported), the perceived warmth and competence of conspiracy spreaders increased in intergroup conflict conditions compared to no‐conflict conditions. While not hypothesized a‐priori, these findings align with the notion that spreading conspiracy theories may benefit one's reputation during intergroup conflict: the negative effects on perceived warmth and competence are attenuated during intergroup conflict. However, conflict intensity did not influence trait impressions.

## STUDY 3

In Study 3 we pitted an intergroup conflict condition against an intergroup cooperation condition to show that when intergroup relations discourage conspiracies, spreading conspiracy theories might decrease the person's competence and warmth.

Study 3 had a 2 × 2 between‐subject design employing the same paradigm as the previous studies. We manipulated both context (cooperation vs. conflict) and the narratives (neutral vs. conspiracy) and hypothesized that conspiracy spreaders are perceived as (1) more dominant than neutral people in general, regardless of context (H3.1); (2) less competent than neutral people in the cooperation condition (H3.2); (3) more competent in the conflict than in the cooperation condition (H3.3); (4) less warm than neutral people (H3.4); (5) less warm in the cooperation than in the conflict condition (H3.5).

### Method

#### Participants

To achieve 80% power (a small‐to‐medium‐sized effect for the interaction, effect size *f* = .15, *α* = .05), a minimum of 351 participants were required.^4^ To be on the safe side, 400 participants from the U.S. were recruited (100 for each condition) via Prolific. Nine participants who failed the attention and manipulation checks were excluded. As a result, 391 participants were eligible (aged from 18 to 78, *M* = 38.4, *SD* = 14.1), including 192 males, 193 females and 6 others.

#### Materials

In addition to the resource conflict scenario, we developed a cooperation scenario. It described how the two tribes had to work together and have been cooperating, for a long time. The conspiracy theory and neutral claim were the same as in other studies.

#### Measurements

The measurements of dominance, competence, warmth and leader‐like qualities were the same as in Study 1 and 2. They had good internal reliability: *α* (dominance) = .79; *α* (leaderlike) = .77; *α* (competence) = .83; *α* (warmth) = .91.

#### Procedure

Participants read the texts and finished the trait‐impression questions.

### Result

#### Descriptive results

The means, standard deviations and correlations are shown in Table 3.

**TABLE 3:** The means, standard deviations and correlations of measured variables (*N* = 391)

	*M*	*SD*	1	2	3	4	5	6	7
1. Gender	1.5	0.5	–						
2. Age	38.4	14.1	.14**	–					
3. Conflict	0.5	0.5	−.04	−.03	–				
4. Narrative	0.5	0.5	−.05	.06	.03	–			
5. Dominance	4.9	0.9	−.01	.08	.01	.21***	–		
6. Leaderlike	5.0	1.0	−.07	−.01	.02	−.12*	.51***	–	
7. Competence	4.8	1.0	−.01	<.01	.07	−.22***	.36***	.64***	–
8. Warmth	4.3	1.2	.03	−.01	.09	−.49***	−.02	.39***	.61***

*Note*: For Gender, ‘male’, ‘female’ and ‘other’ were coded as ‘1’, ‘2’ and ‘3’; for Conflict and Narrative, ‘0’ referred to ‘cooperation’ and ‘neutral’ while ‘1’ referred to ‘conflict’ and ‘conspiracy’.

* *p* < .05.

** *p* < .01.

*** *p* < .001.

#### Hypotheses testing

**FIGURE 4:**
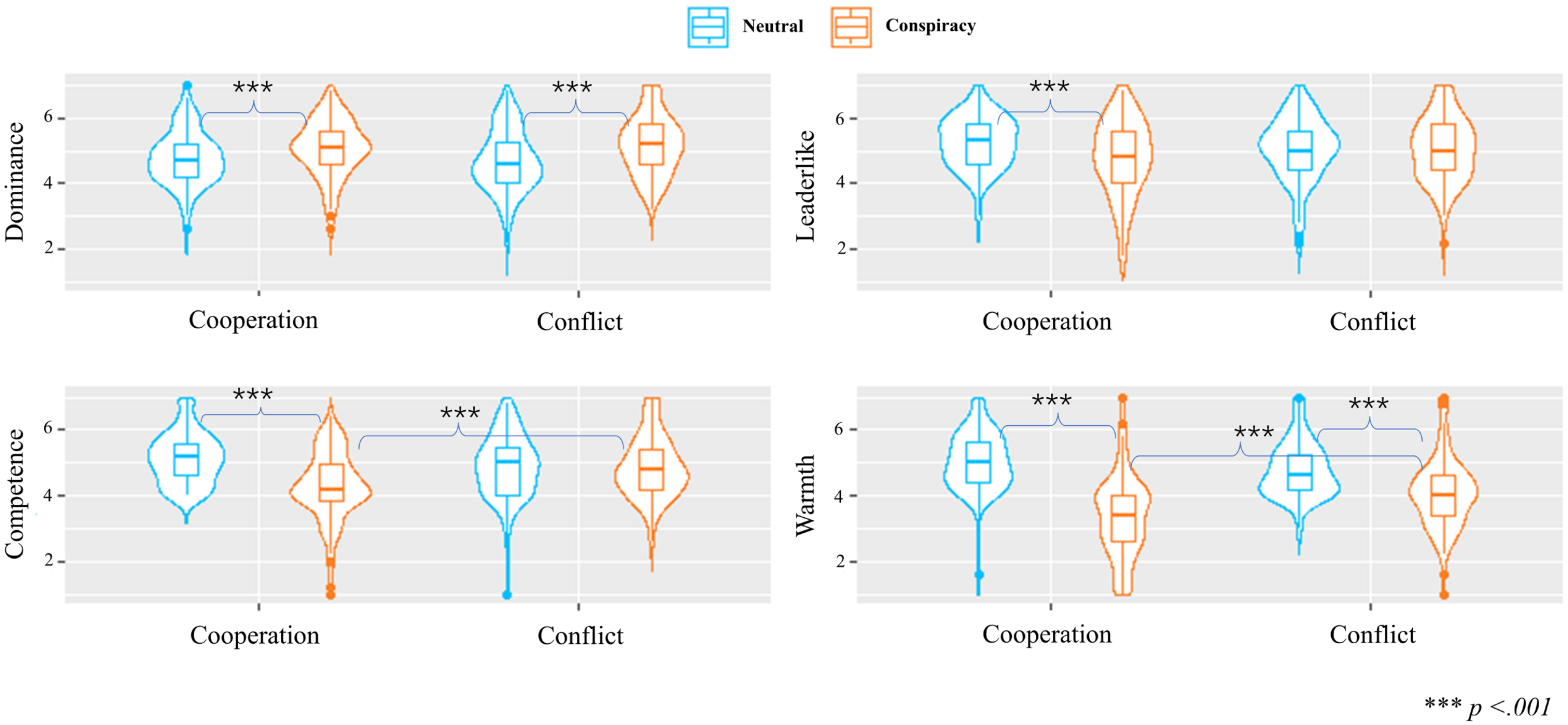
The results of the two‐way ANOVAs.

A series of 2 × 2 ANOVAs were conducted and the results are displayed in Figure 4. For perceived dominance, people considered conspiracy spreaders more dominant (*M* = 5.1, *SD* = 0.9) than the neutral person (*M* = 4.7, *SD* = 0.9), *F*(1, 387) = 18.74, *p* < .001, $\eta_{p}^{2}$ = .05, CI_95%_ [0.01, 0.09]. No significant interaction was found. These results were in line with H3.1.

For perceived competence, conspiracy spreaders (*M* = 4.6, *SD* = 1.0) were perceived as less competent than neutral people (*M* = 5.0, *SD* = 0.9), *F*(1, 387) = 20.58, *p* < .001, $\eta_{p}^{2}$ = .05, CI_95%_ [0.02, 0.10]. A significant interaction was found, *F*(1, 387) = 21.21, *p* < .001, $\eta_{p}^{2}$ = .05, CI_95%_ [0.02, 0.10]. In the cooperation condition, spreaders' competence (*M* = 4.3, *SD* = 1.1) was perceived as lower than neutral people, *M* = 5.2, *SD* = 0.8, *t*(387) = 6.46, *p* < .001 while in the conflict condition, the significant difference in perceived competence between spreaders (*M* = 4.9, *SD* = 0.9) and neutral people, *M* = 4.9, *SD* = 1.0, *t*(387) = −0.09, *p* = 1.000 was not found (H3.2 was supported). The spreader's competence increased in the conflict condition compared to the cooperation condition, *t*(387) = −4.38, *p* < .001, H3.3 was supported. Neutral people's competence did not differ between the two conditions, *t*(387) = 2.16, *p* = .189.

People were perceived as warmer in the conflict condition (*M* = 4.5, *SD* = 1.1) than in the cooperation condition (*M* = 4.2, *SD* = 1.4), *F*(1, 387) = 4.82, *p* = .029, $\eta_{p}^{2}$ = .01, CI_95%_ [0.00, 0.04]. Additionally, spreaders (*M* = 3.7, *SD* = 1.2) were perceived as less warm than neutral people (*M* = 4.9, *SD* = 0.9) *F*(1, 387) = 132.85, *p* < .001, $\eta_{p}^{2}$ = .26, CI_95%_ [0.19, 0.32] (H3.4 was supported). Moreover, the interaction was significant, *F*(1, 387) = 24.09, *p* < .001, $\eta_{p}^{2}$ = .06, CI_95%_ [0.02, 0.11]. Multiple comparisons with Bonferroni adjustment suggested that spreaders' warmth increased in the conflict condition (*M* = 4.1, *SD* = 1.1) compared to the cooperation condition, *M* = 3.3, *SD* = 1.2, *t*(387) = −5.24, *p* < .001, H3.5 was supported. Such significant difference was not found for neutral people in the cooperation (*M* = 5.1, *SD* = 0.9) versus conflict conditions, *M* = 4.8, *SD* = 0.9, *t*(387) = 1.74, *p* = .500.

Neutral people were perceived as more leader‐like (*M* = 5.2, *SD* = 0.9) than spreaders (*M* = 4.9, *SD* = 1.1), *F*(1, 387) = 5.87, *p* = .016, $\eta_{p}^{2}$ = .01, CI_95%_ [0.00, 0.05]. Importantly, a significant interaction was found, *F*(1, 387) = 6.19, *p* = .013, $\eta_{p}^{2}$ = .02, CI_95%_ [0.00, 0.05]. Multiple comparisons with Bonferroni adjustment showed that spreaders in cooperation condition (*M* = 4.8, *SD* = 1.2) were perceived as less leader‐like than neutral people, *M* = 5.3, *SD* = 0.8, *t*(387) = 3.47, *p* = .004. But in the conflict condition, such significant difference between spreaders (*M* = 5.1, *SD* = 1.0) and the neutral people (*M* = 5.0, *SD* = 1.0) was not found, *t*(387) = −0.07, *p* = 1.000.

### Discussion

The results supported all our predictions. In addition, we also found that compared to neutral individuals, conspiracy spreaders were perceived as less leader‐like in the cooperation condition yet no significant difference was found in the conflict condition. The findings of Study 3 are consistent with the broader theoretical idea that spreading conspiracy theories implies a tradeoff for one's social reputation (particularly between dominance versus warmth) and can in some ways be beneficial, especially during an intergroup conflict.

## STUDY 4

The scenarios used in the previous studies were all about a hunter‐gatherer environment which is remote from the actual experience of citizens in modern societies. To increase the external validity of the present research, Study 4 examined participants' impressions of a conspiracy spreader in a modern business setting. It was a 2 × 2 between‐subject design, manipulating both context (cooperation vs. conflict) and the narratives (neutral vs. conspiracy). The hypotheses were the same as in Study 3.

### Method

#### Participants

To achieve 80% power (a small‐to‐medium‐sized effect for the interaction, effect size *f* = .15, *α* = .05), 351 participants were required. Four hundred participants who are working at companies or organizations in the U.S. were recruited, 100 for each condition. After the manipulation check and attention check, 373 participants were eligible (aged from 18 to 85, *M* = 38.7, *SD* = 12.0), including 191 males, 181 females and 1 other.

#### Materials

The vignette is shown in the OSM, which had the same basic structure as in our previous studies but took place in the context of a cyber‐attack in business.

#### Measurements

The measurements were the same as in the previous studies. Again, they had good internal reliability: *α* (dominance) = .84; *α* (leader‐like qualities) = .75; *α* (competence) = .84; and *α* (warmth) = .86.

#### Procedure

Participants read the texts and finished the trait‐impression questions.

### Result

#### Descriptive results

The means, standard deviations and correlations are shown in Table 4.

**TABLE 4:** The means, standard deviations and correlations of measured variables (*N* = 373)

	*M*	*SD*	1	2	3	4	5	6	7
1. Gender	1.5	0.5	–						
2. Age	38.7	12.0	.02	–					
3. Narrative	0.5	0.5	<.01	−.02	–				
4. Conflict	0.5	0.5	−.03	.01	.05	–			
5. Dominance	4.8	0.9	.15**	.07	.13**	.03	–		
6. Leaderlike	5.1	0.9	.15**	.03	−.12*	.11*	.52***	–	
7. Competence	4.9	1.0	.13*	.06	−.23***	.08	.37***	.60***	–
8. Warmth	4.2	1.0	<.01	−0.01	−.42***	.10*	.01	.38***	.60***

*Note*: For Gender, ‘male’, ‘female’ and ‘other’ were coded as ‘1’, ‘2’ and ‘3’; For Conflict and Narrative, ‘0’ referred to ‘cooperation’ and ‘neutral’ while ‘1’ referred to ‘conflict’ and ‘conspiracy’.

* *p* < .05.

** *p* < .01.

*** *p* < .001.

#### Hypotheses testing

People considered conspiracy spreaders more dominant (*M* = 4.9, *SD* = 0.9) than neutral people (*M* = 4.6, *SD* = 1.0), *F*(1, 369) = 6.62, *p* = .011, $\eta_{p}^{2}$ = .02, CI_95%_ [0.00, 0.05]. No interaction was found, *F*(1, 369) = 2.40, *p* = .122, $\eta_{p}^{2}$ < .01, CI_95%_ [0.00, 0.03].

Spreaders (*M* = 4.6, *SD* = 1.0) were perceived as less competent than neutral people (*M* = 5.1, *SD* = 0.9), *F*(1, 369) = 22.90, *p* < .001, $\eta_{p}^{2}$ = .06, CI_95%_ [0.02, 0.11]. A significant interaction was found, *F*(1, 369) = 5.98, *p* = .015, $\eta_{p}^{2}$ = .02, CI_95%_ [0.00, 0.05]. Multiple comparisons with Bonferroni adjustment suggested that in the cooperation condition, spreaders' competence (*M* = 4.4, *SD* = 1.1) was perceived as lower than neutral people, *M* = 5.1, *SD* = 0.9, *t*(369) = 5.11, *p* < .001. However, in the conflict condition, the difference between the spreader (*M* = 4.8, *SD* = 0.9) and neutral person (*M* = 5.1, *SD* = 0.9) was not significant, *t*(369) = 1.68, *p* = .566. The spreader's competence increased in the conflict condition compared to the cooperation condition, *t*(369) = −3.00, *p* = .018. The neutral person's competence did not differ significantly between the two conditions, *t*(369) = 0.45, *p* = 1.000.

For perceived warmth, people were perceived as warmer in the conflict condition (*M* = 4.3, *SD* = 0.8) than in the cooperation condition (*M* = 4.1, *SD* = 1.1), *F*(1, 369) = 5.17, *p* = .024, $\eta_{p}^{2}$ = .01, CI_95%_ [0.00, 0.05]. Conspiracy spreaders (*M* = 3.8, *SD* = 0.9) were perceived as less warm than the neutral people (*M* = 4.6, *SD* = 0.8), *F*(1, 369) = 83.81, *p* < .001, $\eta_{p}^{2}$ = .19, CI_95%_ [0.12, 0.25]. In addition, a significant interaction was found, *F*(1, 369) = 12.67, *p* < .001, $\eta_{p}^{2}$ = .03, CI_95%_ [0.01, 0.08]. Multiple comparisons with Bonferroni adjustment suggested that the conspiracy spreaders' warmth increased in the conflict condition (*M* = 4.1, *SD* = 0.8) compared to the cooperation condition, *M* = 3.5, *SD* = 1.0, *t*(369) = −4.46, *p* < .001. In contrast, for the neutral person, the difference between the cooperation (*M* = 4.7, *SD* = 0.9) and conflict conditions (*M* = 4.6, *SD* = 0.8) was not found significant, *t*(369) = 0.56, *p* = 1.000.

**FIGURE 5:**
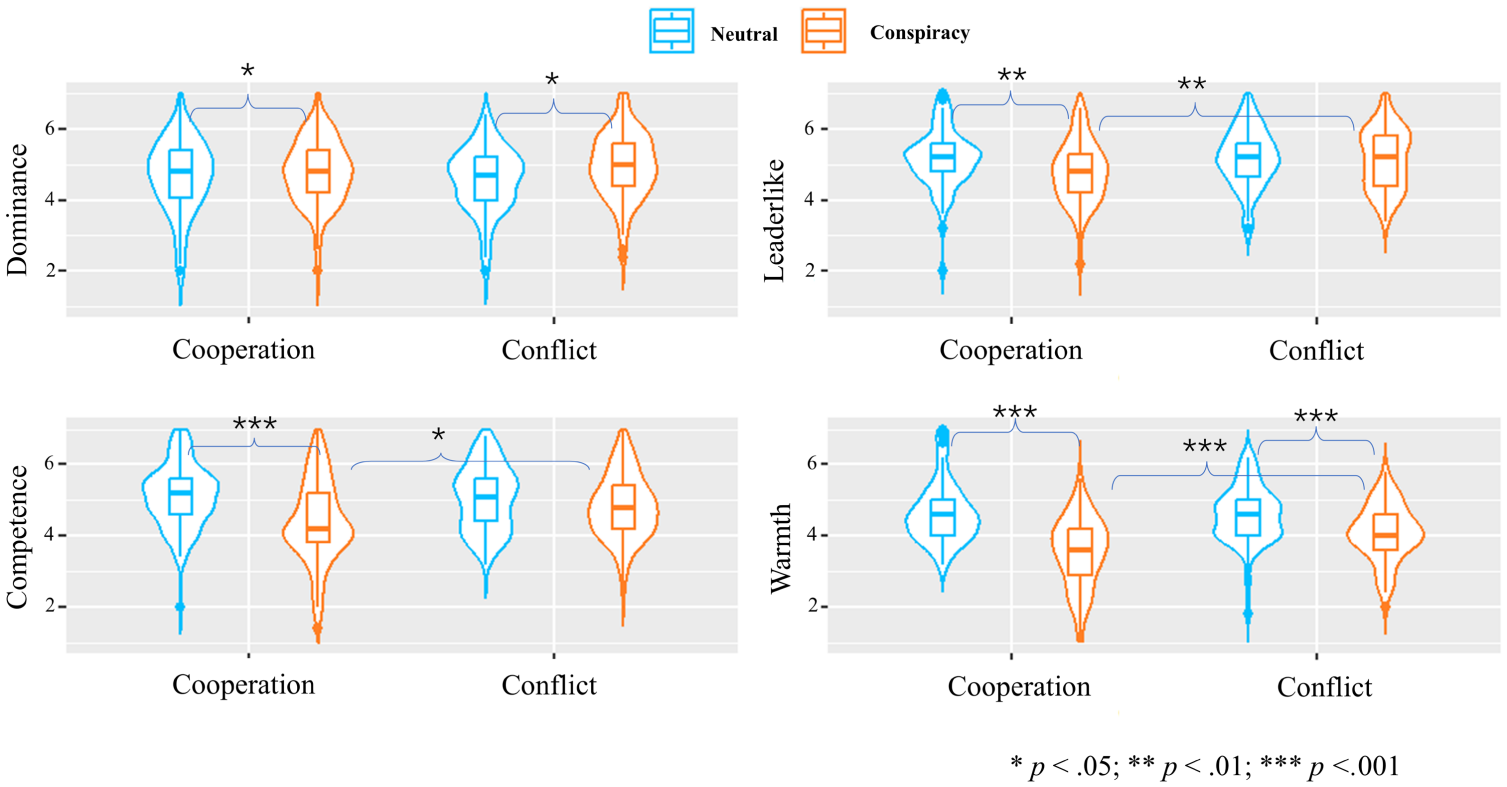
The results of the two‐way ANOVAs.

The neutral person was perceived as more leader‐like (*M* = 5.2, *SD* = 0.8) than the conspiracy spreader (*M* = 5.0, *SD* = 0.9), *F*(1, 369) = 6.36, *p* = .012, $\eta_{p}^{2}$ = .02, CI_95%_ [0.00, 0.05]. In addition, people were perceived as more leader‐like in the conflict condition (*M* = 5.2, *SD* = 0.8) than in the cooperation condition (*M* = 5.0, *SD* = 0.9), *F*(1, 369) = 4.57, *p* = .033, $\eta_{p}^{2}$ = .01, CI_95%_ [0.00, 0.04]. Importantly, a significant interaction was found, *F*(1, 369) = 4.57, *p* = .033, $\eta_{p}^{2}$ = .01, CI_95%_ [0.00, 0.04]. Multiple comparisons with Bonferroni adjustment showed that the conspiracy spreaders in cooperation condition (*M* = 4.8, *SD* = 0.9) was perceived as less leader‐like than neutral people, *M* = 5.2, *SD* = 0.8, *t*(369) = 3.29, *p* = .007. In the conflict condition, the difference between the conspiracy spreader (*M* = 5.1, *SD* = 0.8) and neutral person (*M* = 5.2, *SD* = 0.8) was not found significant, *t*(369) = 0.29, *p* = 1.000. The conspiracy spreader's leader‐like qualities increased in the conflict condition compared to the cooperation condition, *t*(369) = −3.11, *p* = .012. All results are displayed in Figure 5.

### Discussion

Study 4 aimed to replicate the findings and increase the external validity of Studies 1–3 by testing whether similar results could be found in a modern‐world context. Results supported all our predictions and were in line with the findings in Study 3, suggesting that the reputational tradeoff of spreading conspiracy theories could apply to a modern organizational context.

## GENERAL DISCUSSION

### Overview

In five studies, we examined the reputational consequences of spreading conspiracy theories. The pilot study explored how participants perceived conspiracy spreaders (vs. neutral individuals) across three personality traits: dominance, competence and warmth. In an intergroup conflict scenario, conspiracy spreaders were generally perceived as more dominant but less warm than neutral individuals. Study 1 replicated these findings with validated measurements. Study 2 investigated if impressions varied with intergroup conflict intensity (i.e., a resource or physical conflict). Results indicated that conspiracy spreaders were perceived as more competent and warmer in conflict situations compared to no‐conflict, with no impact from conflict intensity. Studies 3 and 4 further validated these results by comparing perceptions of conspiracy spreaders in intergroup cooperation versus conflict situations in both ancestral and modern contexts. Overall, the current studies demonstrated that conspiracy spreaders were consistently perceived as more dominant but less warm than neutral individuals, while negative impressions about their competence and warmth were attenuated during intergroup conflicts. These results provided support for the idea that, besides drawbacks (see also Green, Toribio‐Flórez, & Douglas, 2023; Green, Toribio‐Flórez, Douglas, Brunkow, & Sutton, 2023), there may be reputation benefits for conspiracy spreaders, especially during intergroup conflict.

Impressions of dominance were higher for conspiracy spreaders than for neutral individuals, regardless of the intergroup context. This is consistent with previous studies suggesting a positive relationship between dominance and conspiracy beliefs (Dyrendal et al., 2021; March & Springer, 2019) and provides a possible explanation for this effect: Individuals with a highly dominant personality, or a strong motivation to dominate others, might spread conspiracy theories because it reinforces their dominant reputation. Interestingly, the effect of spreading conspiracy theories on dominance emerged independent of the nature of the intergroup relationship, suggesting that spreading conspiracy theories is a stable behavioral strategy for people to be seen as tough and forceful. While dominance is generally not favored in interpersonal interactions, it is not necessarily seen as a negative trait in an intergroup context; in fact, dominance generally indicates high social rank in groups and people prefer dominant leaders in wartime (Chen et al., 2021; Cheng et al., 2013; Little et al., 2007; Van Vugt & Grabo, 2015).

Unlike dominance, perceived competence of conspiracy spreaders appeared context‐dependent in our studies. In peaceful intergroup settings, conspiracy spreaders may harm potentially beneficial cooperation opportunities with other groups. Therefore, they may be regarded as less competent group members. However, in intergroup conflict situations, conspiracy spreaders demonstrate their ability to detect potential danger, which could aid ingroup survival. While conflict increased the perceived competence of conspiracy spreaders, they were not perceived as more competent than people making neutral statements. Thus, intergroup conflict seemed to mitigate the reputational damage for perceived competence as observed in no‐conflict and cooperation conditions.

Perceived warmth also appeared to be context dependent. Generally seen as less warm than those making neutral statements, conspiracy spreaders faced a reputational drawback, particularly in cooperative situations. Although they were perceived as warmer in the conflict than in no‐conflict or cooperation conditions, they were not seen as warmer than the neutral person. These findings suggest that intergroup conflict mitigates the reputational drawbacks of spreading conspiracy theories (competence and warmth) while not affecting dominance which, to some extent, could be viewed as a reputational benefit.

One may argue that both the conspiracy spreader and the neutral person intended to protect their group, or that the conspiratorial claim in the experiment sounds more plausible than the neutral one because the two groups were conflicting with each other, which may influence the results. According to the ACH, however, the one exposing a conspiracy was protecting the group from a threatening coalition, which is especially important for group survival during intergroup conflict. Spreading conspiracy theories therefore has different reputational consequences. Meanwhile, people's preference for the conspiratorial claim aligns with the notion of ACH, suggesting that individuals exhibit a bias toward conspiracies during intergroup conflict.

Nevertheless, it is important to note that reputational concerns may not be the only reason why people spread conspiracy theories. For example, some people may share conspiracy theories to feel unique (Imhoff & Lamberty, 2017) or generate social engagement (Ren et al., 2023). In addition, some individual differences predict belief in conspiracy theories, such as social dominance orientation, right‐wing authoritarianism and conspiracy mentality (Dyrendal et al., 2021), which may potentially contribute to the spread of conspiracy theories.

### Contributions, limitations and future directions

The present study makes two novel contributions. Firstly, it provides the first empirical evidence, to our knowledge, that spreading conspiracy theories may enhance one's reputation, especially during intergroup conflicts. This suggests a tradeoff between appearing friendly versus tough, with the latter being beneficial in conflict situation. Secondly, our studies provide novel insights suggesting the potential reputational benefits that conspiracy spreaders may obtain, especially in an acute intergroup conflict. The current contribution may hence serve as a framework to further examine the incentives for adopting conspiracy theories and explore interventions that might reduce their proliferation.

However, the present studies have some limitations. We did not consider individual differences, such as paranoid personality or general mistrust, which may influence support for conspiracy narratives (Dyrendal et al., 2021; Freeman et al., 2022). Future studies could explore how people with different personalities (e.g., the Dark Triad, trait hostility and trait aggression) perceive conspiracy spreaders. Additionally, behavioral tasks could examine whether and how spreading conspiracy theories shape the actual interactions between spreaders and perceivers (e.g., are conspiracy spreaders rewarded for their vigilance?).

## CONCLUSION

The presented five studies found that people who spread conspiracy theories were generally seen as more dominant but less warm than the neutral person. Additionally, spreading conspiracy theories was generally associated with reduced perceptions of competence and warmth, yet these negative reputational effects were attenuated during intergroup conflict. Altogether, these findings explain the proliferation of conspiracy theories as communicating them may provide certain beneficial reputational effects, particularly when tensions between groups and organizations are rising.

## AUTHOR CONTRIBUTIONS

**Shen Cao:** Conceptualization; methodology; software; investigation; formal analysis; funding acquisition; visualization; writing – original draft; writing – review and editing; validation; project administration. **Jan‐Willem van Prooijen:** Conceptualization; methodology; supervision; writing – review and editing. **Mark van Vugt:** Conceptualization; methodology; supervision; writing – review and editing.

## CONFLICT OF INTEREST STATEMENT

The authors declare that there are no competing interests.

## Supporting information


Data S1.–S6.

## Data Availability

The data that support the findings of this study are openly available in the Open Science Framework. All data and scripts are available in the OSM at https://osf.io/pr3ua/?view_only=e57edd7b4f5e4459bbdeb2ff50e8f7c9.

## Footnotes

^1^
‘Conspiracy spreaders’ and ‘conspiracy theorists’ are distinct. ‘Conspiracy spreaders’ refer to those who disseminate conspiracy theories, regardless of personal belief. They may use these theories strategically to manipulate others. In contrast, ‘conspiracy theorists’ believe in such theories, but not all share them. In our studies, we use ‘conspiracy spreaders’ to focus on the behavior of spreading conspiracy theories.

^2^
Study 1: https://osf.io/umtes/?view_only=f86080e5686241dcad3a772e003070b4; Study 2: https://osf.io/nbkd7/?view_only=d749bf7a8deb4f68b8041f39e9ac335d; Study 3: https://osf.io/f7ba8/?view_only=e3259fa71b8f4ed7a60f0486debe29ca; Study 4: https://osf.io/shm39/?view_only=f6894328909e4ab4aea82a92799608ec.

^3^
The number of estimated participants was 489 in the pre‐registration, which was calculated with the numerator *df* equaling 3. However, the correct number is 432, a result when the numerator *df* equals 2 (i.e., (2 − 1) × (3−1)).

^4^
The number of estimated participants was 199 in the pre‐registration, which was calculated with *f* equaling 0.2 (with other parameters remaining the same). However, the correct number is 351, a result when *f* equals 0.15. The same deviation existed in Study 4 as well.
