# Supplementary material for: The motivations and reputational consequences of spreading conspiracy theories
Source: Br J Soc Psychol. 2024 Jul 6;64(1):e12784. doi: 10.1111/bjso.12784 (PMC11600388; doi:10.1111/bjso.12784)
Supplement: Supplementary file 1 — Data S1.–S6. [file BJSO-64-0-s001.zip › bjso12784-sup-0006-DataS6.docx]

**Supplementary results**

**Study 1**

We conducted a MANOVA to control for Type I errors. The results with Wilks’s lambda criterion showed that there was a significant effect of the condition, F(5, 382) = 25.37, p < .001.

**Study 2**

We conducted a MANOVA to control for Type I errors. The results with Wilks’s lambda criterion showed that there was a significant main effect of the narrative, F(5, 550) = 29.47, p < .001, and conflict, F(10, 1100) = 1.96, p = .034. The interaction effect was found significant as well, F(10, 1100) = 1.96, p < .001.

**The effects on the perceived commitment**

The α of the commitment to the group was .82. The means, standard deviations, and correlations are shown in Table 1.

**Table 1**

*The means, standard deviations, and correlations of measured variables (N = 560)*

|  | M | SD | 1 | 2 | 3 | 4 | 5 | 6 | 7 | 8 |
| --- | --- | --- | --- | --- | --- | --- | --- | --- | --- | --- |
| 1. gender | 1.5 | 0.5 | - |  |  |  |  |  |  |  |
| 2. age | 42.3 | 13.5 | .14^***^ | - |  |  |  |  |  |  |
| 3. narrative | 0.5 | 0.5 | .04 | -.01 | - |  |  |  |  |  |
| 4. conflict | 1.0 | 0.8 | .04 | -.03 | .02 | - |  |  |  |  |
| 5. dominance | 4.7 | 1.1 | .08 | .11* | .18^***^ | .02 | - |  |  |  |
| 6. leaderlike | 4.8 | 1.0 | -.03 | -.04 | -.01 | .05 | .59^***^ | - |  |  |
| 7. competence | 4.8 | 1.0 | .04 | < .01 | -.18^***^ | .06 | .40^***^ | .63^***^ | - |  |
| 8. warmth | 4.5 | 1.2 | .03 | .03 | -.45^***^ | .02 | .06 | .35^***^ | .59^***^ | - |
| 9. commitment | 5.9 | 1.0 | .10* | .04 | -.08 | -.08 | .18^***^ | .27^***^ | .41^***^ | .31^***^ |

† Note. For Gender, “male", “female”, and “other” were coded as “1”, “2”, and “3”; For Narratives, “0” referred to “neutral” while “1” referred to “conspiracy”; for Conflict, “0” referred to “no-conflict” and “1” referred to “”resource conflict” and “2” referred to “physical conflict”; * p < .05; ** p < .01; *** p < .001

No main effect of conflict was found, F(2, 554) = 2.47, p = .085, η_p_^2^ = .01, CI_95%_[0.00, 0.03], or narrative, F(1, 554) = 3.30, p = .070, η_p_^2^ = .01, CI_95%_[0.00, 0.03]. No interaction was found, F(2, 554) = 2.36, p = .095, η_p_^2^ = .01 CI_95%_[0.00, 0.03].

**Study 3**

The results from MANOVA with Wilks’s lambda criterion suggested that there was a significant main effect of the narrative, *F*(5, 383) = 31.78, *p* < .001, and interaction effect, *F*(5, 383) = 6.74, *p* < .001. The main effect of conflict was not statistically significant, *F*(5, 383) = 1.58, *p* = .166.

**The effects on the perceived commitment**

The α of the commitment to the group was .81. The means, standard deviations, and correlations are shown in Table 2.

**Table 2**

*The means, standard deviations, and correlations of measured variables (N = 391)*

|  | *M* | *SD* | 1 | 2 | 3 | 4 | 5 | 6 | 7 | 8 |
| --- | --- | --- | --- | --- | --- | --- | --- | --- | --- | --- |
| 1. gender | 1.5 | 0.5 | - |  |  |  |  |  |  |  |
| 2. age | 38.4 | 14.1 | .14^**^ | - |  |  |  |  |  |  |
| 3. conflict | 0.5 | 0.5 | -.04 | -.03 | - |  |  |  |  |  |
| 4. narrative | 0.5 | 0.5 | -.05 | .06 | .03 | - |  |  |  |  |
| 5. dominance | 4.9 | 0.9 | -.01 | .08 | .01 | .21^***^ | - |  |  |  |
| 6. leaderlike | 5.0 | 1.0 | -.07 | -.01 | .02 | -.12^*^ | .51^***^ | - |  |  |
| 7. competence | 4.8 | 1.0 | -.01 | <. 01 | .07 | -.22^***^ | .36^***^ | .64^***^ | - |  |
| 8. warmth | 4.3 | 1.2 | .03 | -.01 | .09 | -.49^***^ | -.02 | .39^***^ | .61^***^ | - |
| 9. commitment | 5.8 | 1.1 | .09 | .05 | .05 | -.01 | .20^***^ | .26^***^ | .42^***^ | .29^***^ |

† Note. For Gender, “male", “female”, and “other” were coded as “1”, “2”, and “3”; for Conflict and Narrative, “0” referred to “cooperation” and “neutral” while “1” referred to “conflict” and “conspiracy”; * *p* < .05; ** *p* < .01; *** *p* < .001

For perceived commitment to the group, no main effect of conflict was found, *F*(1, 387) = 1.17, *p* = .281, η_p_^2^ < .01, CI_95%_[0.00, 0.02], nor narrative, *F*(1, 387) = 0.08, *p* = .776, η_p_^2^ < .01, CI_95%_[0.00, 0.01]. No interaction was found, *F*(1, 387) = 0.12, *p* = .733, η_p_^2^ < .01, CI_95%_[0.00, 0.01].

**Study 4**

The results from MANOVA with Wilks’s lambda criterion suggested that there was a significant main effect of the narrative, *F*(5, 365) = 17.21, *p* < .001, and interaction effect, *F*(5, 365) = 2.93, *p* = .013. The main effect of conflict was not found statistically significant, *F*(5, 365) = 0.46, *p* = .807.

**The effects on the perceived commitment**

The α of the commitment to the group was .85. The means, standard deviations, and correlations are shown in Table 3.

**Table 3**

*The means, standard deviations, and correlations of measured variables (N = 373)*

|  | *M* | *SD* | 1 | 2 | 3 | 4 | 5 | 6 | 7 | 8 |
| --- | --- | --- | --- | --- | --- | --- | --- | --- | --- | --- |
| 1. gender | 1.5 | 0.5 | - |  |  |  |  |  |  |  |
| 2. age | 38.7 | 12.0 | .02 | - |  |  |  |  |  |  |
| 3. narrative | 0.5 | 0.5 | < .01 | -.02 | - |  |  |  |  |  |
| 4. conflict | 0.5 | 0.5 | -.03 | .01 | .05 | - |  |  |  |  |
| 5. dominance | 4.8 | 0.9 | .15** | .07 | .13** | .03 | - |  |  |  |
| 6. leaderlike | 5.1 | 0.9 | .15** | .03 | -.12* | .11* | .52*** | - |  |  |
| 7. competence | 4.9 | 1.0 | .13* | .06 | -.23*** | .08 | .37*** | .60*** | - |  |
| 8. warmth | 4.2 | 1.0 | < .01 | -0.01 | -.42*** | .10* | .01 | .38*** | .60*** | - |
| 9. commitment | 5.6 | 1.0 | .06 | .09 | .01 | .13* | .25*** | .42*** | .52*** | .28*** |

† Note. For Gender, “male", “female”, and “other” were coded as “1”, “2”, and “3”; For Conflict and Narrative, “0” referred to “cooperation” and “neutral” while “1” referred to “conflict” and “conspiracy”; * p < .05; ** p < .01; *** p < .001

For perceived commitment to the group, people were perceived as having higher commitment in the conflict condition (*M* = 5.7, *SD* = 0.9) than in the cooperation condition (*M* = 5.4, *SD* = 1.1), *F*(1, 369) = 6.15, *p* = .014, η_p_^2^ = .02, CI_95%_[0.00, 0.05]. No main effect of narrative was found, *F*(1, 369) = 0.02, *p* = .897, η_p_^2^ < .01, CI_95%_[0.00, 0.01]. No interaction was found neither, *F*(1, 369) = 3.13, *p* = .079, η_p_^2^ < .01, CI_95%_[0.00, 0.04].
