# Supplementary material for: The motivations and reputational consequences of spreading conspiracy theories
Source: Br J Soc Psychol. 2024 Jul 6;64(1):e12784. doi: 10.1111/bjso.12784 (PMC11600388; doi:10.1111/bjso.12784)
Supplement: Supplementary file 1 — Data S1.–S6. [file BJSO-64-0-s001.zip › bjso12784-sup-0001-DataS1.zip!/EFA_result.docx]

Exploratory factor analysis (EFA). An EFA was conducted to test whether participants’ scores on The Eight-Factor Structure of Personality Traits loaded on certain common factors. Bartlett’s test of sphericity (Bartlett, 1950) showed that the correlation matrix was not random, χ^2^(153) = 2674.53, p < .001. The Kaiser-Meyer-Olkin (KMO, Kaiser, 1974) measure of sampling adequacy suggested that the relationships among items (KMO = .91) were stronger than the minimum standard for conducting factor analysis (.50). Therefore, the correlation matrix was appropriate for factor analysis. The principal component analysis suggested a two-factor model. The two-factor solution was examined with an orthogonal rotation (varimax). Factor 1 was loaded by 12 items with an eigenvalue of 6.90, accounting for 36% of the total variance; Factor 2 was loaded by 6 items with an eigenvalue of 2.72, accounting for 12% of the total variance. The two-factor solution was sufficient but fit poorly with the data (χ2(118) = 349.96, p < .001). The figure for the principal component analysis and the table for the factor loadings are provided in the appendix. According to the EFA, Factor 1 items (e.g., friendly, trustworthy, sympathetic, helpful, and goodhearted) was conceptually similar to warmth while Factor 2 was similar to dominance (e.g., going along with everything (reversed), dominant, and rousing public sentiment). Thus, although we did not find a factor that represented competence, the results were partially in line with our hypothesis.

Factor loadings for The Eight-Factor Structure of Personality Traits

| Items | Factor1 | Factor2 |
| --- | --- | --- |
| Friendly | 0.71 |  |
| Trustworthy | 0.80 |  |
| Unsympathetic (R) | 0.69 |  |
| Solve_problems | 0.68 | 0.45 |
| Helps_people | 0.86 |  |
| Goodhearted | 0.81 |  |
| Helpful | 0.86 |  |
| Careful | 0.71 |  |
| Do_things_thoroughly | 0.67 |  |
| Careless (R) | 0.75 |  |
| Impulsive (R) | 0.59 | -0.30 |
| Unselfish | 0.61 |  |
| Rouses_public_sentiment |  | 0.52 |
| Dominant |  | 0.71 |
| Go along with everything (R) |  | 0.56 |
| Avoids_difficulties (R) |  | 0.46 |
| Go with the tide (R) |  | 0.46 |
| Complicated |  | 0.30 |
