# Supplementary material for: The motivations and reputational consequences of spreading conspiracy theories
Source: Br J Soc Psychol. 2024 Jul 6;64(1):e12784. doi: 10.1111/bjso.12784 (PMC11600388; doi:10.1111/bjso.12784)
Supplement: Supplementary file 1 — Data S1.–S6. [file BJSO-64-0-s001.zip › bjso12784-sup-0001-DataS1.zip!/materials_pilot.docx]

Conspiracy condition

Please imagine that you are a member of a tribe living in the Amazon Rainforest. Your tribe lives close to another tribe. The two tribes rely on the same resources for their living, but there is not always enough food for both tribes in this territory. So, your tribe is in constant competition with the other tribe. In the long run, only one of the two tribes can probably survive in this environment; the other will either have to leave the territory or run the risk of being disbanded.

Recently, some of your tribe members have died of bites by poisonous snakes. This is strange because there never used to be any poisonous snakes in this part of the Amazon rainforest.
 
During a tribal meeting, Aru, one of the tribal members, stands up and has this to say:

Neutral condition

Please imagine that you are a member of a tribe living in the Amazon Rainforest. Your tribe lives close to another tribe. The two tribes rely on the same resources for their living, but there is not always enough food for both tribes in this territory. So, your tribe is in constant competition with the other tribe. In the long run, only one of the two tribes can probably survive in this environment; the other will either have to leave the territory or run the risk of being disbanded.

Recently, some of your tribe members have died of bites by poisonous snakes. This is strange because there never used to be any poisonous snakes in this part of the Amazon rainforest.

During a tribal meeting, Aru, one of the tribal members, stands up and has this to say:
    

To what extent do you agree with the following description of Aru?

 **Aru is a friendly person.**

- 1- Strongly disagree
- 2- Disagree
- 3- More or less disagree
- 4- Undecided
- 5- More or less agree
- 6- Agree
- 7- Strongly agree

To what extent do you agree with the following description of Aru?

 **Aru is someone one can trust.**

- 1- Strongly disagree
- 2- Disagree
- 3- More or less disagree
- 4- Undecided
- 5- More or less agree
- 6- Agree
- 7- Strongly agree

To what extent do you agree with the following description of Aru?

 **Aru is an unsympathetic person.**

- 1- Strongly disagree
- 2- Disagree
- 3- More or less disagree
- 4- Undecided
- 5- More or less agree
- 6- Agree
- 7- Strongly agree

To what extent do you agree with the following description of Aru?

 **Aru is someone who solves problems.**

- 1- Strongly disagree
- 2- Disagree
- 3- More or less disagree
- 4- Undecided
- 5- More or less agree
- 6- Agree
- 7- Strongly agree

To what extent do you agree with the following description of Aru?

 **Aru is someone who helps people.**

- 1- Strongly disagree
- 2- Disagree
- 3- More or less disagree
- 4- Undecided
- 5- More or less agree
- 6- Agree
- 7- Strongly agree

To what extent do you agree with the following description of Aru?

 **Aru is someone who avoids difficulties.**

- 1- Strongly disagree
- 2- Disagree
- 3- More or less disagree
- 4- Undecided
- 5- More or less agree
- 6- Agree
- 7- Strongly agree

To what extent do you agree with the following description of Aru?

 **Aru is a goodhearted person.**

- 1- Strongly disagree
- 2- Disagree
- 3- More or less disagree
- 4- Undecided
- 5- More or less agree
- 6- Agree
- 7- Strongly agree

To what extent do you agree with the following description of Aru?

 **Aru is a helpful person.**

- 1- Strongly disagree
- 2- Disagree
- 3- More or less disagree
- 4- Undecided
- 5- More or less agree
- 6- Agree
- 7- Strongly agree

To what extent do you agree with the following description of Aru?

 **Aru is a dominant person.**

- 1- Strongly disagree
- 2- Disagree
- 3- More or less disagree
- 4- Undecided
- 5- More or less agree
- 6- Agree
- 7- Strongly agree

To what extent do you agree with the following description of Aru?

 **Aru is a careful person.**

- 1- Strongly disagree
- 2- Disagree
- 3- More or less disagree
- 4- Undecided
- 5- More or less agree
- 6- Agree
- 7- Strongly agree

To what extent do you agree with the following description of Aru?

 **Aru is someone who does things thoroughly.**

- 1- Strongly disagree
- 2- Disagree
- 3- More or less disagree
- 4- Undecided
- 5- More or less agree
- 6- Agree
- 7- Strongly agree

To what extent do you agree with the following description of Aru?

 **Aru is a careless person.**

- 1- Strongly disagree
- 2- Disagree
- 3- More or less disagree
- 4- Undecided
- 5- More or less agree
- 6- Agree
- 7- Strongly agree

To what extent do you agree with the following description of Aru?

 **Aru is someone who rouses public sentiment.**

- 1- Strongly disagree
- 2- Disagree
- 3- More or less disagree
- 4- Undecided
- 5- More or less agree
- 6- Agree
- 7- Strongly agree

To what extent do you agree with the following description of Aru?

 **Aru is an impulsive person.**

- 1- Strongly disagree
- 2- Disagree
- 3- More or less disagree
- 4- Undecided
- 5- More or less agree
- 6- Agree
- 7- Strongly agree

To what extent do you agree with the following description of Aru?

 **Aru is someone who behaves unselfishly.**

- 1- Strongly disagree
- 2- Disagree
- 3- More or less disagree
- 4- Undecided
- 5- More or less agree
- 6- Agree
- 7- Strongly agree

To what extent do you agree with the following description of Aru?

 **Aru is someone who goes with the tide.**

- 1- Strongly disagree
- 2- Disagree
- 3- More or less disagree
- 4- Undecided
- 5- More or less agree
- 6- Agree
- 7- Strongly agree

To what extent do you agree with the following description of Aru?

 **Aru goes along with everything.**

- 1- Strongly disagree
- 2- Disagree
- 3- More or less disagree
- 4- Undecided
- 5- More or less agree
- 6- Agree
- 7- Strongly agree

To what extent do you agree with the following description of Aru?

 **Aru is a complicated person.**

- 1- Strongly disagree
- 2- Disagree
- 3- More or less disagree
- 4- Undecided
- 5- More or less agree
- 6- Agree
- 7- Strongly agree
