# Supplementary material for: The motivations and reputational consequences of spreading conspiracy theories
Source: Br J Soc Psychol. 2024 Jul 6;64(1):e12784. doi: 10.1111/bjso.12784 (PMC11600388; doi:10.1111/bjso.12784)
Supplement: Supplementary file 1 — Data S1.–S6. [file BJSO-64-0-s001.zip › bjso12784-sup-0002-DataS2.zip!/materials_study1.docx]

Conspiracy condition

Please imagine that you are a member of a tribe living in the Amazon Rainforest. Your tribe lives close to another tribe. The two tribes rely on the same resources for their living, but there is not always enough food for both tribes in this territory. So, your tribe is in constant competition with the other tribe. In the long run, only one of the two tribes can probably survive in this environment; the other will either have to leave the territory or run the risk of being disbanded.

Recently, some of your tribe members have died of bites by poisonous snakes. This is strange because there never used to be any poisonous snakes in this part of the Amazon rainforest.
 
During a tribal meeting, Aru, one of the tribal members, stands up and has this to say:

Neutral condition

Please imagine that you are a member of a tribe living in the Amazon Rainforest. Your tribe lives close to another tribe. The two tribes rely on the same resources for their living, but there is not always enough food for both tribes in this territory. So, your tribe is in constant competition with the other tribe. In the long run, only one of the two tribes can probably survive in this environment; the other will either have to leave the territory or run the risk of being disbanded.

Recently, some of your tribe members have died of bites by poisonous snakes. This is strange because there never used to be any poisonous snakes in this part of the Amazon rainforest.

During a tribal meeting, Aru, one of the tribal members, stands up and has this to say:
 

How do you think of Aru?

| Dominance | 1-  Not at all | 2 | 3 | 4-Moderate | 5 | 6 | 7-  Very much |
| --- | --- | --- | --- | --- | --- | --- | --- |
| I think Aru is dominant. |  |  |  |  |  |  |  |
| I think Aru is forceful. |  |  |  |  |  |  |  |
| I think Aru is influential. |  |  |  |  |  |  |  |
| I think Aru is powerful. |  |  |  |  |  |  |  |
| I think Aru is self-assured. |  |  |  |  |  |  |  |

How well does each item fit your image of Aru?

| Leaderlike | 1-  Not at all | 2 | 3 | 4 -Moderate | 5 | 6 | 7  -Very much |
| --- | --- | --- | --- | --- | --- | --- | --- |
| Emphasizes goals |  |  |  |  |  |  |  |
| Coordinates groups |  |  |  |  |  |  |  |
| Proposes solutions |  |  |  |  |  |  |  |
| Specifies problems |  |  |  |  |  |  |  |
| Talks frequently |  |  |  |  |  |  |  |

End of Block: Perceived leaderlike

Start of Block: face

How do you think of Aru?

| Competence | 1  -Not at all | 2 | 3 | 4  -Neutral | 5 | 6 | 7  -Very much |
| --- | --- | --- | --- | --- | --- | --- | --- |
| Aru has much potential. |  |  |  |  |  |  |  |
| Aru performs very well at a number of things. |  |  |  |  |  |  |  |
| Aru is talented. |  |  |  |  |  |  |  |
| Aru is not very competent. |  |  |  |  |  |  |  |
| Aru deals poorly with challenges. |  |  |  |  |  |  |  |
| Please select "Not at all" |  |  |  |  |  |  |  |

I think that Aru

| Warmth | 1  -Not at all | 2 | 3 | 4  -Neutral | 5 | 6 | 7  -Very much |
| --- | --- | --- | --- | --- | --- | --- | --- |
| Is helpful and unselfish with others. |  |  |  |  |  |  |  |
| Has a forgiving nature. |  |  |  |  |  |  |  |
| Can be cold and aloof. |  |  |  |  |  |  |  |
| Is considerate and kind to almost everyone. |  |  |  |  |  |  |  |
| Likes to cooperate with others. |  |  |  |  |  |  |  |

I think that

| Commitment | 1  -Not at all | 2 | 3 | 4  -Neutral | 5 | 6 | 7  -Very much |
| --- | --- | --- | --- | --- | --- | --- | --- |
| Aru would like to work for our tribe. |  |  |  |  |  |  |  |
| Aru dislikes being a member of our tribe. |  |  |  |  |  |  |  |
| Aru would rather belong to the other tribe. |  |  |  |  |  |  |  |
| Aru thinks our tribe has little to be proud of. |  |  |  |  |  |  |  |
| Aru has little respect for our tribe. |  |  |  |  |  |  |  |
