# Supplementary material for: The motivations and reputational consequences of spreading conspiracy theories
Source: Br J Soc Psychol. 2024 Jul 6;64(1):e12784. doi: 10.1111/bjso.12784 (PMC11600388; doi:10.1111/bjso.12784)
Supplement: Supplementary file 1 — Data S1.–S6. [file BJSO-64-0-s001.zip › bjso12784-sup-0004-DataS4.zip!/materials_study3.docx]

Conspiracy-conflict

Please imagine that you are a member of a tribe living in the Amazon Rainforest. Your tribe lives close to another tribe. The two tribes rely on the same resources for their living, but there is not always enough food for both tribes in this territory. Your tribe is in constant competition with the other tribe. In the long run, only one of the two tribes can probably survive in this environment; the other will either have to leave the territory or run the risk of being disbanded.

Recently, some of your tribe members have died of bites by poisonous snakes. This is strange because there never used to be any poisonous snakes in this part of the Amazon rainforest.
 
During a tribal meeting, Aru, one of the tribal members, stands up and has this to say:

Conspiracy-cooperation

Please imagine that you are a member of a tribe living in the Amazon Rainforest. Your tribe lives close to another tribe. The two tribes rely on the same resources for their living, but there is not always enough food for both tribes in this territory. Your tribe is constantly working together with the other tribe in obtaining and sharing food. In the long run, only when the two tribes work closely together can they obtain enough food to survive in this environment; then the tribes will have to leave the territory or run the risk of being disbanded.

Recently, some of your tribe members have died of bites by poisonous snakes. This is strange because there never used to be any poisonous snakes in this part of the Amazon rainforest.
 
During a tribal meeting, Aru, one of the tribal members, stands up and has this to say:

Neutral-conflict

Please imagine that you are a member of a tribe living in the Amazon Rainforest. Your tribe lives close to another tribe. The two tribes rely on the same resources for their living, but there is not always enough food for both tribes in this territory. Your tribe is in constant competition with the other tribe. In the long run, only one of the two tribes can probably survive in this environment; the other will either have to leave the territory or run the risk of being disbanded.

Recently, some of your tribe members have died of bites by poisonous snakes. This is strange because there never used to be any poisonous snakes in this part of the Amazon rainforest.

During a tribal meeting, Aru, one of the tribal members, stands up and has this to say:

     

Neutral-cooperation

Please imagine that you are a member of a tribe living in the Amazon Rainforest. Your tribe lives close to another tribe. The two tribes rely on the same resources for their living, but there is not always enough food for both tribes in this territory. Your tribe is constantly working together with the other tribe in obtaining and sharing food. In the long run, only when the two tribes work closely together can they obtain enough food to survive in this environment; then the tribes will have to leave the territory or run the risk of being disbanded.

Recently, some of your tribe members have died of bites by poisonous snakes. This is strange because there never used to be any poisonous snakes in this part of the Amazon rainforest.

During a tribal meeting, Aru, one of the tribal members, stands up and has this to say:

           
