# Supplementary material for: The motivations and reputational consequences of spreading conspiracy theories
Source: Br J Soc Psychol. 2024 Jul 6;64(1):e12784. doi: 10.1111/bjso.12784 (PMC11600388; doi:10.1111/bjso.12784)
Supplement: Supplementary file 1 — Data S1.–S6. [file BJSO-64-0-s001.zip › bjso12784-sup-0005-DataS5.zip!/materials_study4.docx]

**Conspiracy + Conflict**

Please imagine that you are a devoted member of an e-commerce company. Your company is in cutthroat competition with another company named Loco, vying for market supremacy. Now, at the most critical juncture of this competition, both companies have implemented numerous strategies to hinder each other's expansion. In the long run, only one of the two companies can stay on top of the market. The other is likely to face deteriorating financial conditions or even the threat of bankruptcy and restructuring.

Three days ago, your company hosted a crucial commercial event, which has consistently accounted for a significant portion of its annual profits. However, an unforeseen incident transpired, causing immense distress and uncertainty. The company's IT system, which had always functioned seamlessly in the past, unexpectedly experienced a failure just hours before the event was scheduled to kick off. Customers remained unable to access the online shop's website for three hours beyond the promised start time. This resulted in significant profit losses and customer complaints. None of your colleagues have any insight into the root cause of this system breakdown.

During the emergency meeting of your company, Jack, a colleague of you, stood up and had this to say:

**Conspiracy + Cooperation**

Please imagine that you are a devoted member of an e-commerce company. Your company is in a fruitful cooperation with another company named Loco, jointly vying for market supremacy. Now, at the most critical juncture of this cooperation, both companies have implemented numerous strategies to help each other's expansion. In the long run, it's clear that by helping each other, both companies can stay on top of the market.

Three days ago, your company hosted a crucial commercial event, which has consistently accounted for a significant portion of its annual profits. However, an unforeseen incident transpired, causing immense distress and uncertainty. The company's IT system, which had always functioned seamlessly in the past, unexpectedly experienced a failure just hours before the event was scheduled to kick off. Customers remained unable to access the online shop's website for three hours beyond the promised start time. This resulted in significant profit losses and customer complaints. None of your colleagues possess any insight into the root cause of this system breakdown.

During the emergency meeting of your company, Jack, a colleague of you, stood up and had this to say:

**Neutral + Conflict**

Please imagine that you are a devoted member of an e-commerce company. Your company is in cutthroat competition with another company named Loco, vying for market supremacy. Now, at the most critical juncture of this competition, both companies have implemented numerous strategies to hinder each other's expansion. In the long run, only one of the two companies can stay on top of the market. The other is likely to face deteriorating financial conditions or even the threat of bankruptcy and restructuring.

Three days ago, your company hosted a crucial commercial event, which has consistently accounted for a significant portion of its annual profits. However, an unforeseen incident transpired, causing immense distress and uncertainty. The company's IT system, which had always functioned seamlessly in the past, unexpectedly experienced a failure just hours before the event was scheduled to kick off. Customers remained unable to access the online shop's website for three hours beyond the promised start time. This resulted in significant profit losses and customer complaints. None of your colleagues have any insight into the root cause of this system breakdown.

During the emergency meeting of your company, Jack, a colleague of you, stood up and had this to say:

**Neutral + Cooperation**

Please imagine that you are a devoted member of an e-commerce company. Your company is in a fruitful cooperation with another company named Loco, jointly vying for market supremacy. Now, at the most critical juncture of this cooperation, both companies have implemented numerous strategies to help each other's expansion. In the long run, it's clear that by helping each other, both companies can stay on top of the market.

Three days ago, your company hosted a crucial commercial event, which has consistently accounted for a significant portion of its annual profits. However, an unforeseen incident transpired, causing immense distress and uncertainty. The company's IT system, which had always functioned seamlessly in the past, unexpectedly experienced a failure just hours before the event was scheduled to kick off. Customers remained unable to access the online shop's website for three hours beyond the promised start time. This resulted in significant profit losses and customer complaints. None of your colleagues possess any insight into the root cause of this system breakdown.

During the emergency meeting of your company, Jack, a colleague of you, stood up and had this to say:
